# Atheroprotective effects of methotrexate via the inhibition of YAP/TAZ under disturbed flow

**DOI:** 10.1186/s12967-019-02135-8

**Published:** 2019-11-15

**Authors:** Dandan Liu, Hang Lv, Qi Liu, Yanli Sun, Shenglong Hou, Lu Zhang, Mengyue Yang, Baihe Han, Gang wang, Xuedong Wang, Wenjuan Du, Honggang Nie, Ruoxi Zhang, Xingtao Huang, Jingbo Hou, Bo Yu

**Affiliations:** 1 Division Department of Cardiology Organization, The Second Affiliated Hospital of Harbin Medical University, Harbin, China; 2 Key Laboratories of the Education Ministry for Myocardial Ischemia Mechanisms and Treatment, Harbin, China; 3 Division of Cardiovascular Surgery, The Second Affiliated Hospital of Harbin Medical University, Harbin, China

**Keywords:** Methotrexate, Human umbilical vein endothelial cells, YAP/TAZ, Shear stress, AMP-dependent kinase

## Abstract

**Background:**

Atherosclerosis preferentially develops in regions of disturbed flow (DF). Emerging evidence indicates that yes-associated protein (YAP) and transcriptional co-activator with PDZ-binding motif (TAZ), which are both effectors of the Hippo pathway, sense different blood flow patterns and regulate atherosclerotic lesions. We previously found that methotrexate (MTX) reduces in-stent neoatherosclerosis, decreases the plaque burden, and has an effect on local fluid shear stress. Here, we investigated the atheroprotective effect of MTX under DF and the mechanisms underlying these properties.

**Methods:**

Human umbilical vein endothelial cells (HUVECs) were subjected to biomechanical stretch using a parallel-plate flow system and treated with or without MTX at therapeutically relevant concentrations. Additionally, an extravascular device was used to induce DF in the left common carotid artery of C57BL/6 mice, followed by treatment with MTX or 0.9% saline. The artery was then assessed histopathologically after 4 weeks on a Western diet.

**Results:**

We observed that MTX significantly inhibited DF-induced endothelial YAP/TAZ activation. Furthermore, it markedly decreased pro-inflammatory factor secretion and monocyte adhesion in HUVECs but had no effect on apoptosis. Mechanistically, AMPKa1 depletion attenuated these effects of MTX. Accordingly, MTX decreased DF-induced plaque formation, which was accompanied by YAP/TAZ downregulation in vivo.

**Conclusions:**

Taken together, we conclude that MTX exerts protective effects via the AMP-dependent kinase (AMPK)-YAP/TAZ pathway. These results provide a basis for the prevention and treatment of atherosclerosis via the inhibition of YAP/TAZ.

## Background

Atherosclerosis is currently the leading cause of mortality worldwide [1, 2]. Despite advances in disease-modifying and biological therapy for the disease, specific strategies aimed at retarding its development are lacking, and the knowledge of whether individual drugs offer vascular protection is limited. Atherosclerotic lesions develop in the arteries at sites of disturbed flow (DF) and shear stress plays a critical role in plaque location and progression [3–5]. Recent research has demonstrated that systemic inhibition of the Hippo pathway effectors yes-associated protein (YAP) and transcriptional co-activator with PDZ-binding motif (TAZ) attenuates the development of atherosclerotic lesions induced by DF [6]. YAP/TAZ responds to haemodynamic forces and transduces mechanical signals into chemical signals [7]. DF promotes YAP/TAZ activation and dephosphorylation, and the dephosphorylated form of YAP is translocated from the cytoplasm to the nucleus to up-regulate target genes including cysteine-rich angiogenic inducer 61 (*CYR61*) and connective tissue growth factor (*CTGF*), and to stimulate pro-inflammatory gene expression, thereby increasing monocyte attachment and infiltration, which contribute to atherogenesis [6, 8].

Several studies have demonstrated that long-term low-dose methotrexate (MTX) therapy in rheumatoid arthritis is associated with reduced cardiovascular disease and cardiovascular mortality [9, 10]. Likewise, MTX-treated animals show reduced rates of lipid-rich intima [11]. MTX increases the intracellular accumulation of adenosine monophosphate (AMP) and 5-aminoimidazole-4-carboxamide ribonucleotide (AICAR), which activates AMP-activated protein kinase (AMPK) [12, 13]. AMPK plays a role in promoting YAP phosphorylation at Ser127, which phosphorylates YAP to induce its cytoplasmic localization and proteasomal degradation; therefore, AMPK activation results in YAP phosphorylation and inactivation [14–16]. We hypothesised that the AMPK pathway mediates YAP/TAZ functional inactivation and the atheroprotective effects of MTX.

Therefore, in this study, the contribution of MTX to atheroprotective effects and the signalling mechanism underlying such protective effects against DF were evaluated.

## Materials and methods

### Materials and reagents

Human umbilical vein endothelial cells (HUVECs) (ScienCell Research Laboratories, Carlsbad, CA, USA) were grown in endothelial cell medium supplemented with 5% foetal bovine serum (FBS), 100 units/mL penicillin, and 100 μg/mL streptomycin. THP1 cells were obtained from the FuDan IBS Cell Center (Shanghai, China) and maintained in RPMI medium 1640 supplemented with 10% (vol/vol) FBS and 1% penicillin–streptomycin. Trizol and CELLTRACE Violet were obtained from Invitrogen (Carlsbad, CA, USA). iScript gDNA Clear cDNA Synthesis Kit and SsoFast EvaGreen Supermix were obtained from Bio-RAD. Antibodies against p-YAP (Ser-127), YAP, TAZ, ICAM1, VCAM1, AMPK, p-AMPK, LATS1, p-LATS1 (Thr 1079), and β-actin used for western blotting were all purchased from Cell Signaling Technology (CST, Danvers, MA, USA). Antibodies against YAP and p-YAP used for immunofluorescence staining were purchased from CST and antibodies against YAP/TAZ used for immunofluorescence staining were purchased from Abcam (Cambridge, UK). A directly conjugated Alexa Fluor 488-CD31 antibody was purchased from Biolegend (San Diego, CA). An Annexin V-FITC Apoptosis Detection Kit was purchased from Becton–Dickinson (Franklin Lakes, NJ, USA). Small interfering RNAs against YAP (siYAP) and AMPKα1 (siAMPKα1), as well as the negative control molecule (siNC), were purchased from RiboBio (Guangzhou, China). Methotrexate (in vitro) and atorvastatin were purchased from Aladdin (Shanghai, China). MTX (in vivo) was purchased from Pfizer (Bentley WA, Australia).

### Cell culture and treatment

HUVECs were cultured in endothelial cell medium supplemented with 5% FBS at 37 °C in a 5% CO_2_ humidified atmosphere and passaged every 2–3 days. Cells within seven passages were used for the in vitro study. For MTX treatment, before HUVECs were exposed to shear stress for the indicated durations (0, 1, 10, and 24 h), they were preincubated with MTX (100 nM) for 48 h [17]; the same procedure was used for atorvastatin (1 μM) [18].

### Shear stress experiments

A parallel-plate flow system was used to apply shear stress to HUVECs cultured in flow channels following previously established methods [19]. HUVECs were seeded onto fibronectin-coated glass slides and grown. The static (STA) treatment, as a control, unidirectional shear stress (USS), and DF were applied to HUVECs for 1, 10, or 24 h with a shear stress of 12 dyn/cm^2^ and 0 ± 4 dyn/cm^2^.

### Western blotting

Western blotting was used to detect the expression of target proteins in HUVECs. Briefly, equal amounts of total cell lysates were blotted onto a PVDF membrane and incubated with primary antibodies against YAP (1:1000), p-YAP (1:1000; Ser 127), TAZ (1:1000), ICAM-1 (1:1000), VCAM-1 (1:1000), p-AMPK (1:1000), AMPK (1:1000), and β-actin (1:1000) overnight at 4 °C. Membranes were washed three times and then incubated with peroxidase-conjugated secondary antibody (1:5000) for 1 h at 37 °C. Immunoreactive bands were detected by electrochemiluminescence (ECL) and exposure to X-ray film. Protein levels were quantified using scanning densitometry (ImageJ, National Institutes of Health, Bethesda, MD, USA). All data were obtained from three independent experiments.

### Monocyte adhesion assay

THP1 monocytes were maintained in RPMI medium 1640 containing 10% (vol/vol) FBS and 0.1% penicillin–streptomycin. THP-1 monocytes were labelled with CellTrace Violet and resuspended at 1 × 10^6^ cells/mL in 1640 medium. The adhesion assay was performed by adding the labelled THP-1 cells to HUVECs for 1 h at room temperature. After removing the unbound cells by two washes with 1640 medium, the THP-1 cells attached to HUVECs were fixed with 4% (wt/vol) PFA for 10 min, and adhered THP1 cells were measured using a fluorescence microscope at 50× magnification.

### Immunofluorescence

For in vitro experiments, HUVECs subjected to shear stress for 10 h were fixed with 4% (wt/vol) PFA for 10 min. The cells were permeabilised with 0.3% triton X-100 and blocked with 5% (wt/vol) BSA in PBS for 30 min, which was followed by incubation with the primary antibody against YAP, p-YAP (1:100) at 4 °C overnight. For arterial samples, frozen sections were embedded with optimal cutting temperature (OCT) compound, permeabilized, blocked, and incubated with primary antibodies against YAP/TAZ (1:100) at 4 °C overnight. After the incubation with primary antibodies, goat anti-rabbit IgG antibodies were used (1:100) as the secondary antibodies. The endothelial areas were identified in the anti-CD31 stained image, with anti-CD31 used as an endothelial marker. Nuclei were counterstained with DAPI. Images were obtained under a confocal microscope.

### Apoptosis assays

The FITC Annexin V Apoptosis Detection Kit I was used to quantitatively determine the percentage of HUVECs undergoing apoptosis according to the manufacturer’s instructions. After 10 h of shear stress, cells were harvested with trypsin (Beyotime Biotechnology, Haimen, China) and washed twice with cold PBS. Cells were washed twice with cold PBS and resuspended in 100 mL of binding buffer at a concentration of 1 × 10^6^ cells/mL. Then, 5 µL of FITC Annexin V and 5 µL of propidium iodide were added to the cell suspension, followed by incubation at 25 °C for 15 min in the dark with gentle vortexing for double staining. Then, 400 µL of 1× Binding Buffer was added to each sample and immediately analysed by fluorescence-activated cell sorting (FACS) using a flow cytometer (BD Biosciences, San Diego, CA, USA). The data were analysed using FlowJo (FlowJo LLC, Ashland, OR, USA).

### siRNA transfection

HUVECs were transfected with serum-free media containing 50 µM siRNA using the X-treme siRNA Transfection Reagent (RiboBio, Guangzhou, China) in accordance with the manufacturer’s instructions. The transfection reagent was incubated for 15 min at room temperature before it was added to HUVECs and incubated for an additional 24 h. After another 48 h with MTX, the HUVECs on slides were used for the shear stress experiment. The following targeted siRNAs were synthesised by RiboBio: siRNA-AMPKa1 (GATCCATCATATAGTTCAA) and siRNA-YAP1 (CCACCAAGCTAGATAAAGA).

### qRT-PCR analyses

Total RNA was isolated from HUVECs using TRIzol according to the manufacturer’s instructions. First-strand cDNA was generated from 1 µg of RNA using the iScript gDNA Clear cDNA Synthesis Kit. qRT-PCR experiments were performed using the SsoFast EvaGreen Supermix. Glyceraldehyde 3-phosphate dehydrogenase (*GAPDH*) was used as a standard control for normalisation. Each cDNA sample was run in triplicate. qPCR data were analysed using the 2^−∆∆CT^ cycle threshold method. Table 1 presents all primers used for qRT-PCR.

**Table 1:** Primers for qRT-PCR

Gene	Forward	Reverse
*AMPK*-*α*	TGATGTTGTAGTGACACCATTTAC	GAAGATGAGGGAAAGAATTAAGGG
*CTGF*	ACCGACTGGAAGACACGTTTG	CCAGGTCAGCTTCGCAAGG
*CYR*-*61*	TGAAGCGGCTCCCTGTTTT	CGGGTTTCTTTCACAAGGCG
*VCAM1*	CAGTAAGGCAGGCTGTAAAAGA	TGGAGCTGGTAGACCCTCG
*ICAM1*	TTGGGCATAGAGACCCCGTT	GCACATTGCTCAGTTCATACACC
*IL*-*6*	CTCAATATTAGAGTCTCAACCCCCA	GAGAAGGCAACTGGACCGAA
*IL*-*8*	CCACCGGAGCACTCCATAAG	GATGGTTCCTTCCGGTGGTT
*YAP*	GCTACAGTGTCCCTCGAACC	CCGGTGCATGTGTCTCCTTA
*TAZ*	ATCCCCAACAGACCCGTTTC	GAACGCAGGCTTGCAGAAAA
*GAPDH*	ACGGATTTGGTCGTATTGGGC	TTGACGGTGCCATGGAATTTG

### Animals

Male C57BL/6 mice weighing at least 18 g were purchased from the Second Affiliated Hospital of the Harbin Medical University Laboratory Animal Centre and fed a Western-type diet. All animal care was conducted in accordance with the “Principles of Animal Care” (Ethical and Animal Welfare Committee of Heilongjiang Province, China) and were approved by the ethics review board of Harbin Medical University. Briefly, after anaesthetisation with isoflurane, DF was altered by cast placement in the left common carotid artery, as previously described [20]. Animals were randomly allocated to two groups; one group was treated with MTX at 1 mg/kg/week [21] and the other group received an equal volume of 0.9% saline by weekly intraperitoneal injections. The treatments began on the day of cast placement. Four weeks later, all animals were sacrificed. The carotid artery was embedded in OCT compound and 7-μm cryosections were prepared for haematoxylin–eosin (H&E) staining, Masson staining, and immunofluorescence.

### Statistical analysis

Data are expressed as mean ± SD. Student’s *t*-tests were used to evaluate differences between two groups and a one-way ANOVA with Tukey’s post hoc test was used for multiple groups. GraphPad Prism version 7.0 was used for analyses and p < 0.05 was considered statistically significant.

## Results

### Haemodynamic regulation of the activation and nuclear localisation of YAP/TAZ in HUVECs

**Fig. 1:**
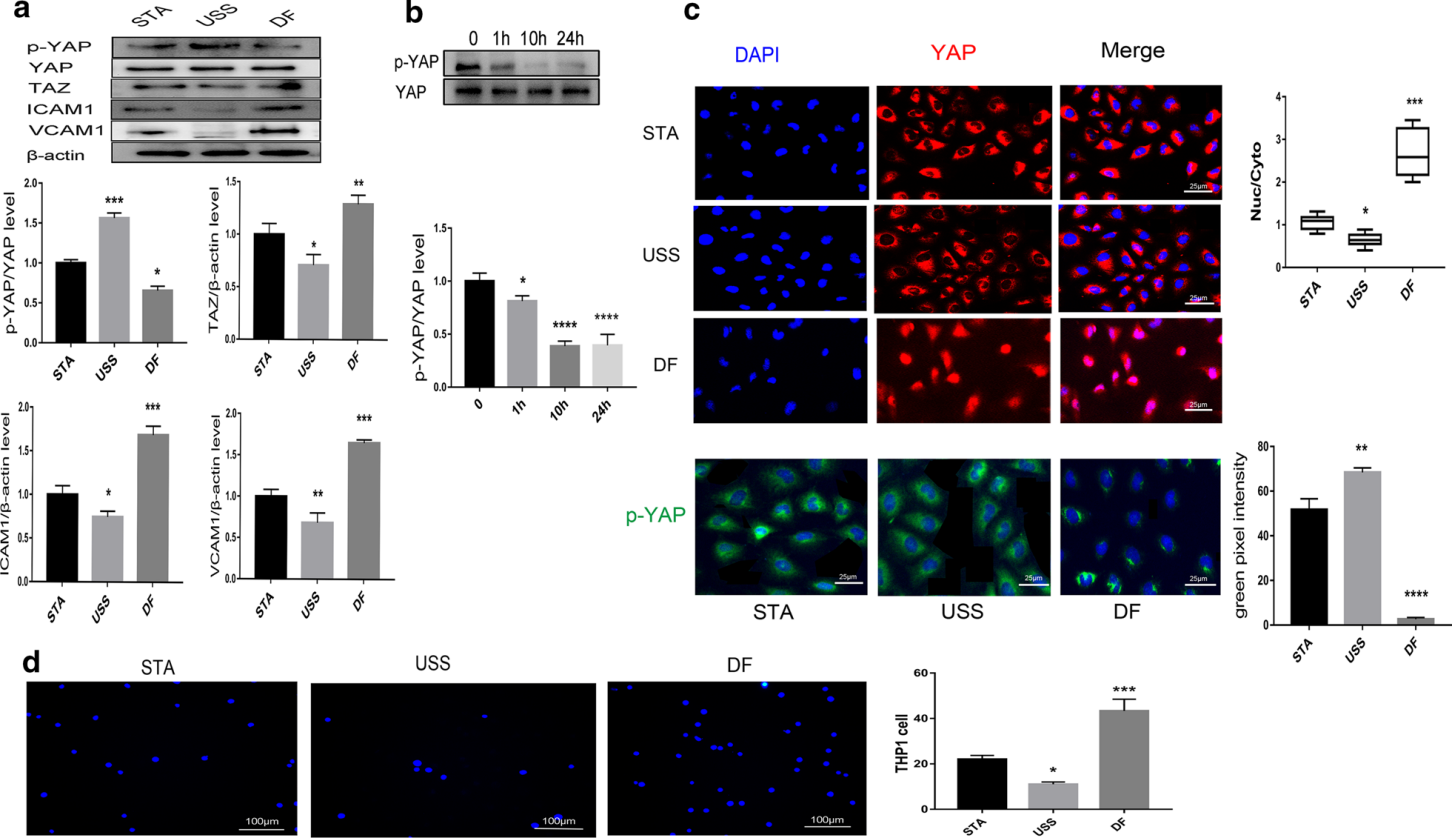
Haemodynamic regulation of the activation and nuclear localisation of YAP/TAZ in HUVECs. **a** Western blot analysis of VCAM1, ICAM1, and YAP/TAZ subjected to USS (unidirectional shear stress), disturbed flow (DF), or static (STA) control. The histograms show quantitative protein levels. Data are presented as mean ± SD from triplicate experiments, *p < 0.05, **p < 0.01, ***p < 0.001 vs. STA. **b** Western blot analysis of p-YAP and YAP in HUVECs subjected to DF for the indicated time. *p < 0.05, ****p < 0.0001 vs. control. **c** Immunofluorescence staining of p-YAP and YAP in HUVECs subjected to different flow patterns. YAP (red) and p-YAP (green) were visualised by immunostaining; nuclei were counterstained with propidium iodide (PI, blue). The graphic data are the YAP intensity ratios (nuclear/cytoplasmic) of cells randomly selected from three independent experiments. *p < 0.05, ***p < 0.001 vs. STA. **d** Representative images of THP1 cell (blue) adhesion to HUVECs for different flows (×50)

To investigate the roles of YAP/TAZ in HUVECs under different shear forces, cultured HUVECs were exposed to USS, DF, or STA for 10 h. We measured the protein expression levels of p-YAP (Ser127), total-YAP/TAZ, and adhesion molecules by western blotting. Our results demonstrated that DF leads to a significant decrease in p-YAP (Ser127) (Fig. 1a, b) and marked increases in ICAM1, VCAM1, and TAZ expression (Fig. 1a). IF staining showed that DF results in YAP/TAZ nuclear localisation, whereas increased YAP/TAZ cytoplasmic retention was observed in HUVECs subjected to USS. In addition, the levels of YAP phosphorylation were also examined by IF; we found that DF markedly decreased p-YAP (Fig. 1c), consistent with the western blotting results (Fig. 1a).

**Fig. 2:**
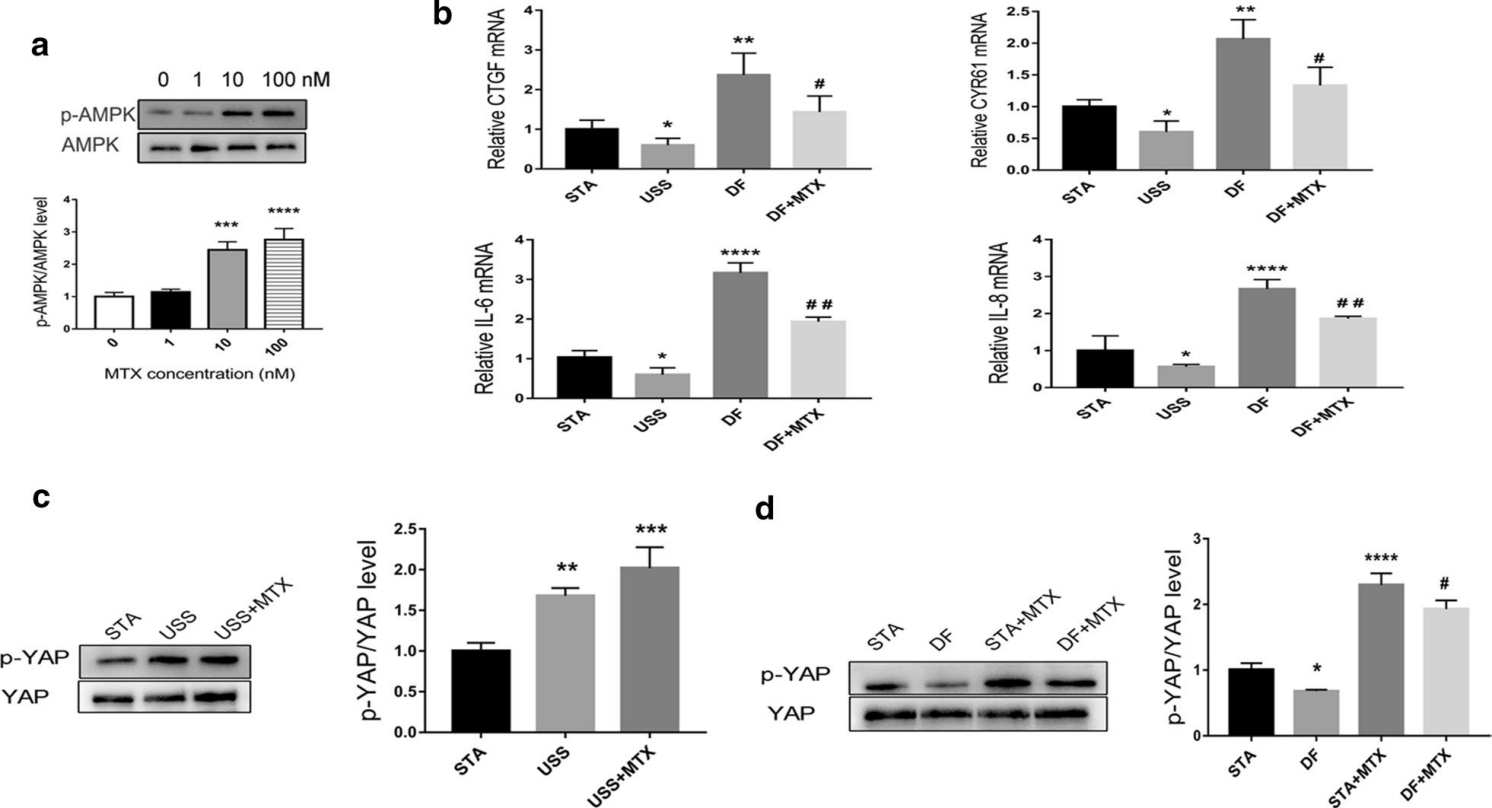
MTX treatment increases the level of p-YAP and alleviates the expression of YAP target genes and inflammatory factors. **a** HUVECs were treated with MTX (0–100 nM) and p-AMPK was quantified after 48 h by a western blot analysis. ***p < 0.001, ****p < 0.0001 vs. control. **b** qRT-PCR analysis of YAP/TAZ target genes and inflammatory factors for different flows. Results are presented as mean ± SD from three independent experiments from each group. *p < 0.05, **p < 0.01, ****p < 0.0001 vs. STA (static), and ^#^p < 0.05, ^##^p < 0.01 vs. DF. **c** HUVECs were treated with or without MTX (100 nM) under USS, and p-YAP (Ser127) was quantified by western blot analysis. **p < 0.01,***p < 0.001 vs. STA. **d** HUVECs were treated with or without MTX under STA or DF, and p-YAP (Ser127) was quantified by western blot analysis. *p < 0.05, ****p < 0.0001 vs. STA; ^#^p < 0.05 vs. STA + MTX

DF promoted the transactivation activity of YAP/TAZ and significantly increased the expression of the YAP/TAZ target genes *CYR61* and *CTGF* (Fig. 2b). In contrast, USS enhanced p-YAP (Figs. 1a, 2c) and reduced YAP/TAZ target gene expression (Fig. 2b). Moreover, biomechanical stretch mediates the ability of HUVECs to recruit monocytes. We used a cell adhesion assay and found an increased number of THP1 monocytes attached to HUVECs under DF (Fig. 1d), which was accompanied by the up-regulation of adhesion molecules (Fig. 1a). These results indicated that DF leads to YAP dephosphorylation and activation, whereas USS results in YAP phosphorylation and inactivation.

### MTX phosphorylates AMPK and alleviates DF-induced proinflammatory cytokine expression in HUVECs

HUVECs were treated with MTX (0–100 nM) and p-AMPK levels were quantified after 48 h by western blotting. After treatment with MTX for 48 h, p-AMPK in HUVECs cultured in static conditions increased in a dose-dependent manner, with a maximum level at 100 nM (Fig. 2a). The concentration was consistent with conventional low-dose therapeutic dosing for patient plasma [22].

To establish whether MTX alters proinflammatory cytokine expression under haemodynamic forces, qRT-PCR was used to analyse levels of proinflammatory cytokine genes and YAP/TAZ target genes. Indeed, we observed higher levels of *IL*-*6*, *IL*-*8*, *CYR61*, and *CTGF* under DF rather than USS in HUVECs, and MTX treatment significantly suppressed their expression (Fig. 2b).

To determine whether the low-dose MTX induced changes downstream of AMPK, we examined the protein expression levels of p-YAP (Ser127) under different flow conditions. The results demonstrated that DF, but not USS, led to a significant decrease in YAP phosphorylation (Fig. 2c, d). However, when we compared the effects of MTX treatment vs. control under USS, we did not observe a significant change in YAP phosphorylation (Fig. 2c). MTX treatment led to a marked increase in p-YAP expression under DF or STA, with the latter leading to higher p-YAP expression after MTX treatment (Fig. 2d).

### MTX inhibits DF-induced YAP/TAZ activation and exerts atheroprotective effects, whereas silencing AMPKα reverses these effects

**Fig. 3:**
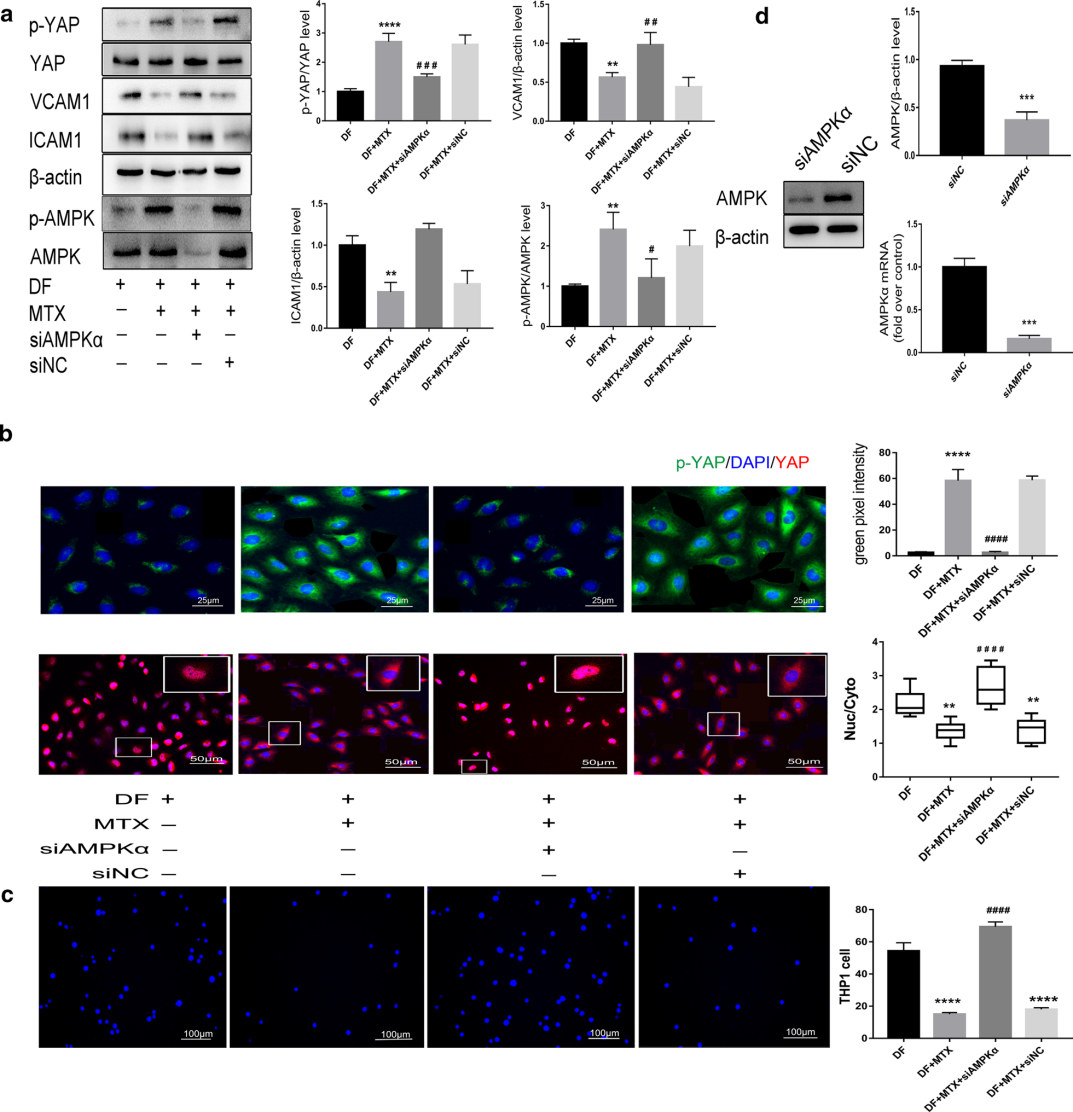
MTX suppresses YAP activation, whereas silencing AMPKα abolishes this effect. **a** Representative western blots of p-YAP (Ser127), YAP, p-AMPK, total AMPK (AMPK), β-actin, ICAM-1, and VCAM-1 in HUVECs after each treatment. Values represent the mean ± SD from three independent experiments from each group. **p < 0.01, ****p < 0.0001 vs. DF; ^##^p < 0.01, ^###^p < 0.001 vs. DF + MTX. **b** Immunofluorescence staining of p-YAP and YAP in HUVECs. p-YAP (green) and YAP (red) were visualised by immunostaining; nuclei were counterstained with propidium iodide (PI, blue). Boxed regions are enlarged images. The graphic data are the YAP intensity ratios (nuclear/cytoplasmic) of cells randomly selected from three independent experiments. **p < 0.01 vs. DF, ^####^p < 0.0001 vs. DF + MTX. MTX decreased YAP nuclear localisation under DF; siAMPKα counteracted the effects. **c** Representative images of THP1 cell (blue) adhesion to the differently-treated HUVECs under DF. The graphic data show quantification of THP1 cells from three independent experiments. **d** AMPK, as measured by qRT-PCR (n = 3) and western blotting (n = 3), in HUVECs after transfection with siNC or siAMPKα for 36 h. ***p < 0.001, ****p < 0.0001 vs. siNC

We next explored whether MTX mediates YAP/TAZ inactivation in HUVECs under biomechanical stretch by immunofluorescence and western blotting. A western blot analysis revealed that MTX significantly up-regulated the expression of p-YAP and p-AMPK and markedly decreased the levels of ICAM1 and VCAM1 under DF (Fig. 3a). We also observed that total YAP was constitutively expressed at high levels in the cytoplasm and that monocyte adhesion was reduced after MTX (100 nM) treatment under DF (Fig. 3b, c). These data suggest that MTX mediates YAP inhibition and reduces monocyte–HUVEC interactions, contributing, at least in part, to anti-atherogenic effects.

Because YAP can be phosphorylated by AMPK [23], we further explored the role of AMPK in this MTX-induced protective effect and determined whether MTX-mediated YAP phosphorylation is AMPK-dependent. AMPKα depletion by siRNA (Fig. 3d) promoted YAP nuclear translocation and nearly abolished MTX-mediated YAP phosphorylation (Fig. 3a) and p-YAP cytoplasmic localisation (Fig. 3b), indicating a critical role of AMPK in mediating MTX-induced YAP inactivation. Following AMPK knockdown in HUVECs, both the levels of monocyte adhesion proteins and the augmentation of monocyte adhesion were significantly increased under DF (Fig. 3a, c).

### Silencing YAP reduces adhesion molecule expression under DF

**Fig. 4:**
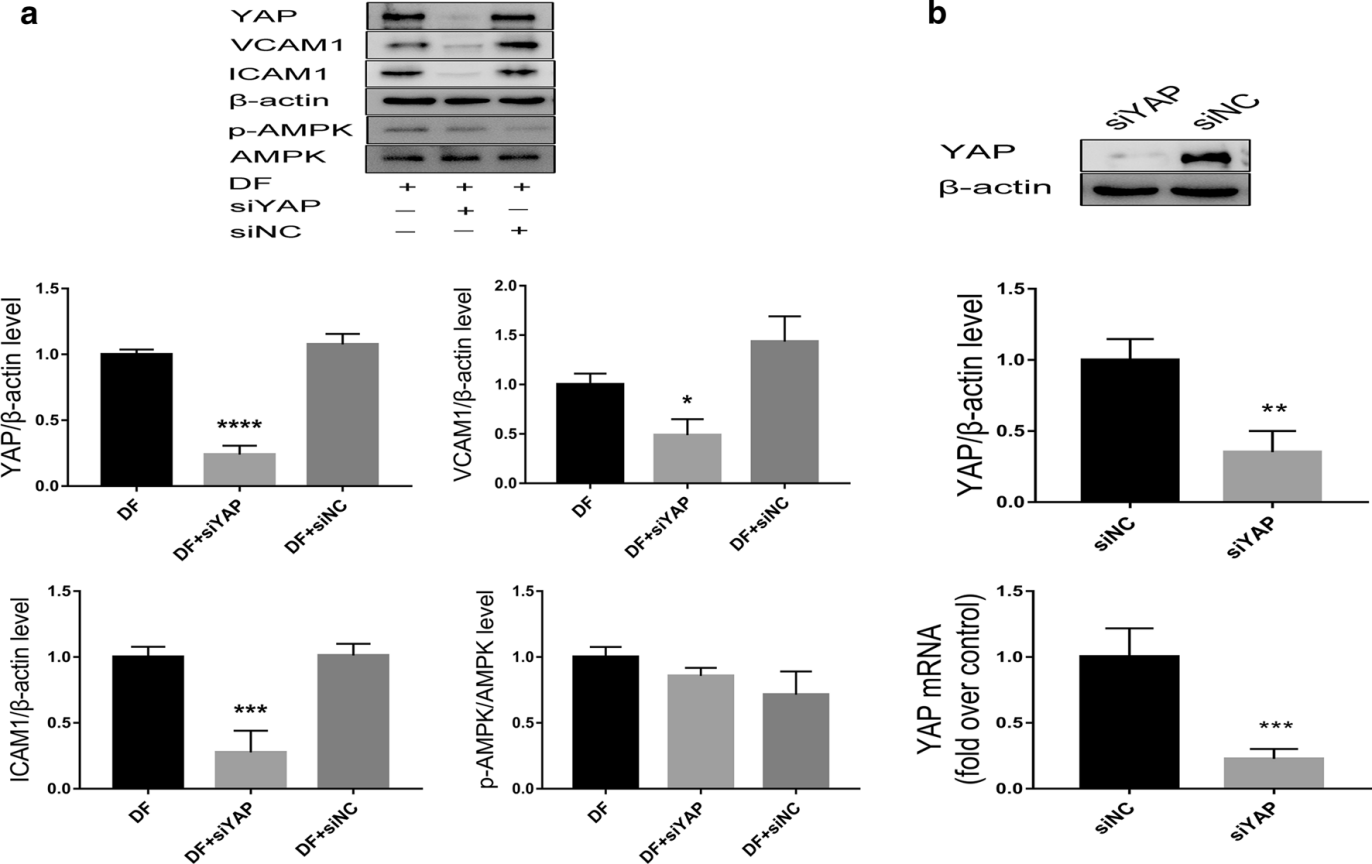
Silencing YAP reduces adhesion molecule expression but does not alter p-AMPK expression under DF. **a** HUVECs were transfected with siYAP or siNC, subjected to DF for 24 h, and evaluated by western blotting to detect YAP, VCAM1, ICAM1, p-AMPK, and AMPK. *p < 0.05, ***p < 0.001, ****p < 0.0001 vs. DF. **b** YAP, as measured by qRT-PCR and western blotting, in HUVECs after transfection with siNC or siAMPKα for 36 h. All data are shown as mean ± SD of triplicate experiments and are representative of three independent experiments. **p < 0.01, ****p < 0.0001 vs. siNC

To further confirm the association between YAP and DF-induced proinflammatory responses, siRNAs against YAP were used to examine the interactions among YAP, adhesion molecules, and AMPK. Western blotting results demonstrated that knocking down YAP significantly inhibited the expression of ICAM1 and VCAM1 but did not alter p-AMPK protein levels under DF (Fig. 4a, b), indicating that AMPK acts upstream of YAP, consistent with previous results [14], and that YAP depletion might contribute to atherogenesis.

### Effects of MTX on DF-induced apoptosis and YAP phosphorylation

**Fig. 5:**
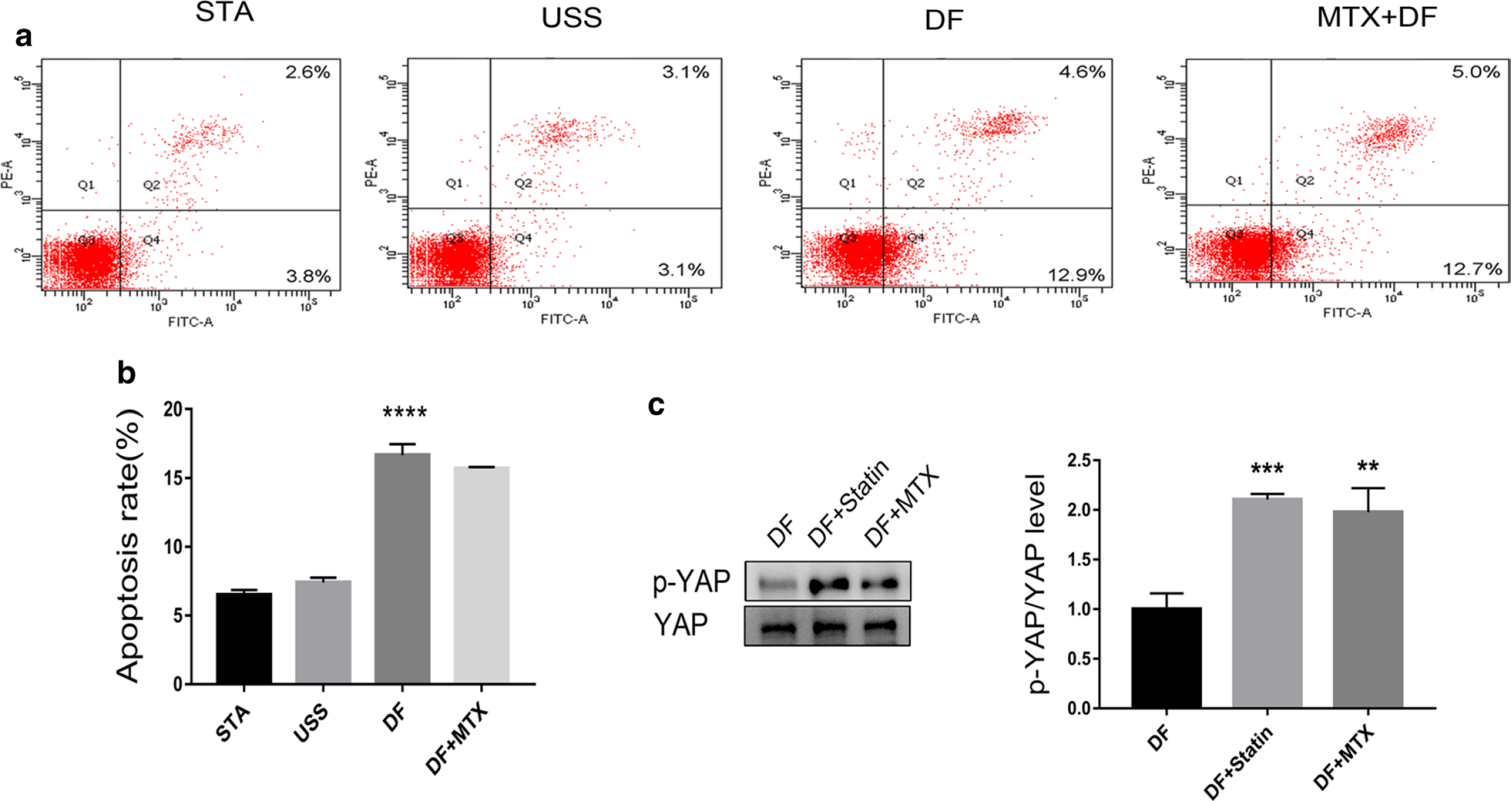
MTX has no effect on DF-induced HUVEC apoptosis and atorvastatin promotes YAP phosphorylation. **a** HUVECs were incubated with MTX for 48 h and subjected to biomechanical stretch for another 10 h. Apoptosis in HUVECs is shown as flow cytometry dot plots. **b** As quantified by flow cytometry, DF increased the total apoptosis rate compared to that under static (STA) conditions. However, treatment with MTX did not reduce apoptosis. The percentage of apoptotic cells is presented as the mean ± SD from triplicate experiments. ****p < 0.0001 vs. STA. **c** Western blotting results (left panel) and quantitative data (right panel) for the protein levels of p-YAP are presented. Values are presented as mean ± SD from three independent experiments. **p < 0.01, ***p < 0.001 vs. DF

Impaired endothelial function comprises an early stage of atherosclerosis. In this study, flow cytometry was used to assay cell apoptosis under DF. Compared to that with STA, the application of DF, but not USS, significantly increased the total apoptosis rate. However, treatment with MTX did not reduce apoptosis (Fig. 5a, b).

Using western blot analyses, we detected comparable levels of p-YAP expression in HUVECs treated with MTX and the widely-used atherosclerotic drug atorvastatin under DF. Our results demonstrated that the exposure of cells to DF in the presence of MTX (100 nM) or atorvastatin (1 μM) resulted in the hyperphosphorylation of YAP. There was no statistically significant difference in the level of p-YAP expression between MTX and statin treatments (Fig. 5c).

### MTX treatment decreases DF-induced plaque formation in vivo

**Fig. 6:**
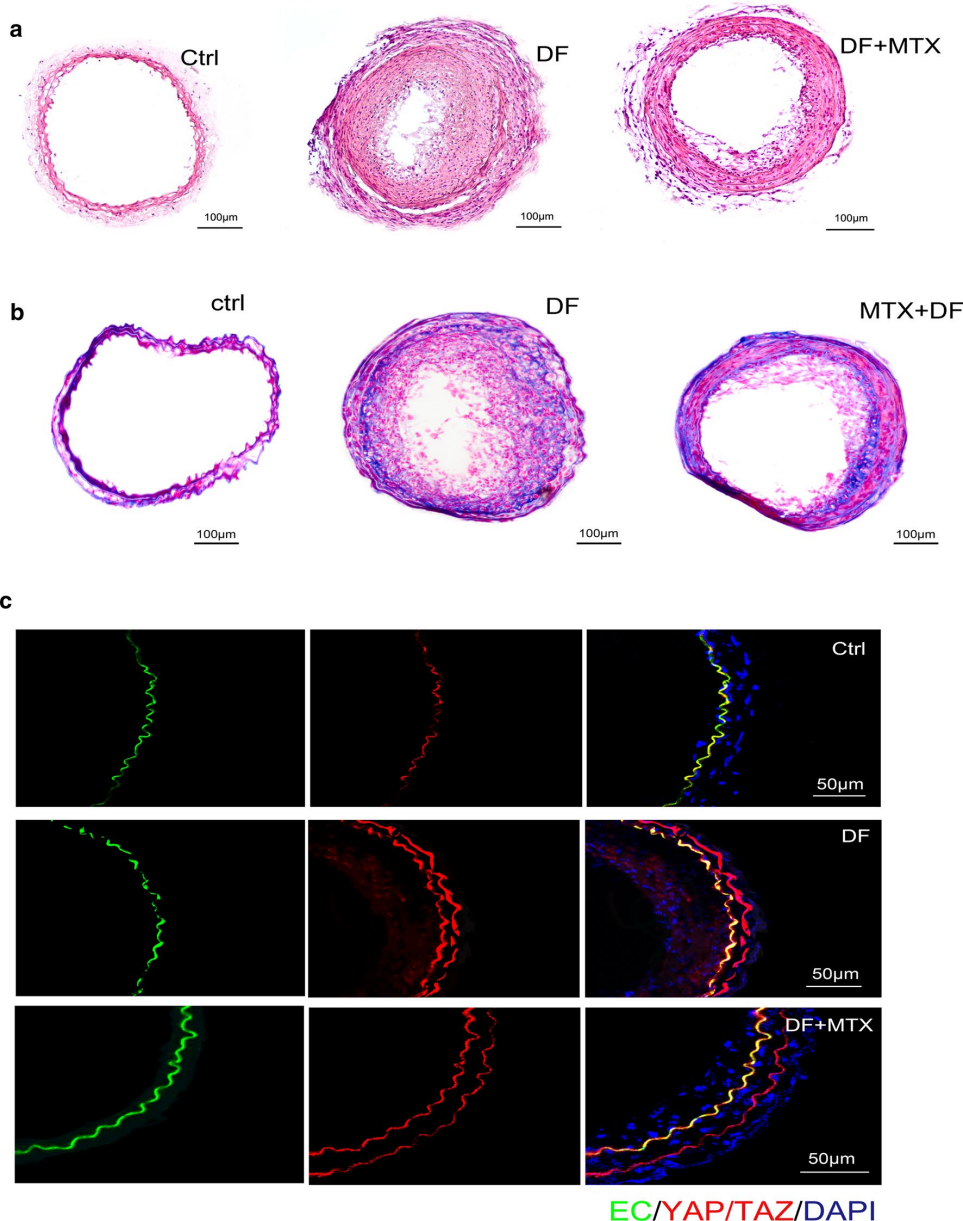
MTX-treated animals present with a lower plaque burden and lower relative YAP/TAZ expression levels in DF regions. **a**, **b** Representative H&E and Masson-stained normal left carotid artery (n = 3) sections from the upstream region of the cast (0.5 mm proximal from the cast) where DF was induced after 0.9% saline treatment for 4 weeks (n = 7) and sections after treatment with MTX (n = 8). **c** Representative immunofluorescence image of the normal left carotid artery treated with 0.9% saline or MTX. Sections were stained with YAP/TAZ (red) and DAPI (Blue). Sections are stained with anti-CD31 as an endothelial marker (green). MTX-treated animals showed lower relative YAP/TAZ expression levels in DF regions

To identify the potential anti-arteriosclerosis effects of low-dose MTX in vivo, a cast was placed around the left common carotid artery to induce DF. After exposure to a Western diet for 4 weeks, apparent atherosclerotic lesions were induced in the DF region (Fig. 6a). YAP/TAZ staining was remarkable in the intima, the media layer, and the intimal hyperplastic plaque of the atherosclerotic section (Fig. 6b). In contrast, treating mice with MTX resulted in a significant reduction in atherosclerotic plaque development (Fig. 6a, b) and relative YAP/TAZ expression (Fig. 6c). Moreover, the lumen in the DF region was larger than that with 0.9% saline treatment (Fig. 6a).

## Discussion

**Fig. 7:**
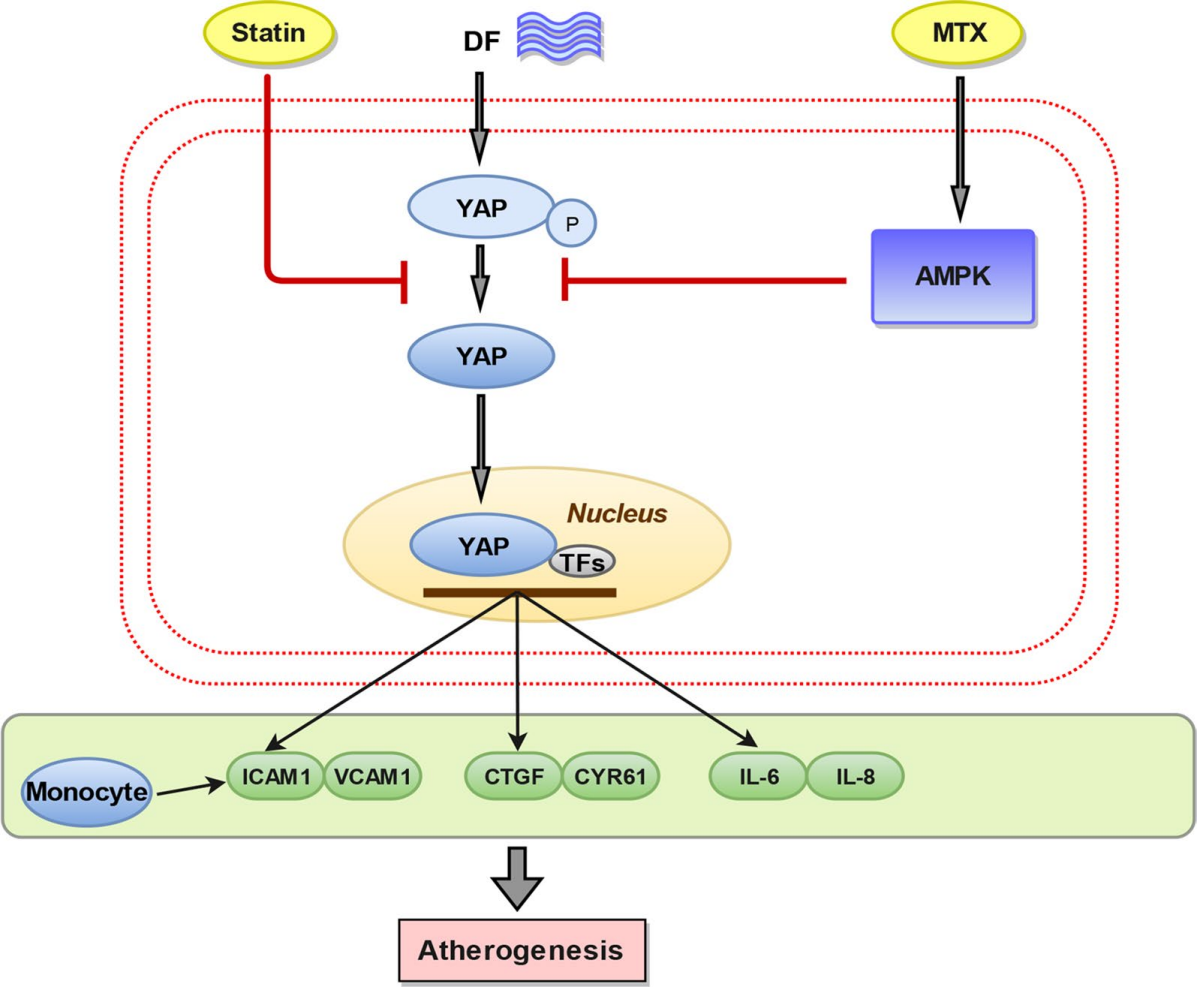
Schematic diagram of the possible mechanism underlying methotrexate (MTX)-mediated protection of HUVECs against disturbed flow (DF) injury via the activation of AMPK-YAP/TAZ signalling

Atherosclerosis is considered an inflammatory disease [24, 25]. Recently, MTX has attracted increasing attention due to its vasculoprotective and anti-inflammatory effects. In this study, we show that the MTX-YAP/TAZ pathway has a profound effect on the development of DF-induced atherosclerosis (Fig. 7). Our results demonstrated that DF leads to YAP activation, a decrease in YAP phosphorylation (Fig. 1a, b) and a marked increase in total YAP expression (Fig. 6c), in accordance with previous results [6, 8], and that the atheroprotective effect of MTX is mediated by the inhibition of YAP activity and up-regulation of p-YAP (Ser127). MTX markedly decreased pro-inflammatory factor secretion and monocyte adhesion under DF (Figs. 2b, 3a, c), which in turn contributed to atherogenesis. These beneficial effects were reversed upon silencing endogenous AMPKα (Fig. 3a, b). Furthermore, MTX-treated mice not only exhibited a significant reduction in plaque development but also lower YAP/TAZ expression under DF (Fig. 6a, c). Taken together, these results indicate that YAP/TAZ may serve as important regulators of atherosclerosis and that MTX might have atheroprotective effects via the inactivation of YAP/TAZ. Accordingly, MTX/AMPK/YAP represents a promising therapeutic target that reduces the expression of atherogenic factors in HUVECs.

A crucial event in atherosclerosis is endothelial dysfunction resulting from haemodynamic forces [26]. HUVECs and human coronary artery endothelial cells, which express abundant YAP, are constantly exposed to mechanical forces generated by the blood flow [27]. YAP/TAZ, which are key factors in the pathophysiology of the cardiovascular system [28], sense a highly diverse range of mechanical cues and translate these cues into specific biochemical signals. YAP located in the nucleus under DF promotes endothelial cell ICAM1 and VCAM1 expression for monocyte adherence, which is correlated with key pathogenic events in atherosclerosis such as endothelial thickening and the recruitment of monocytes, which eventually turn into plaques [7]. Therefore, both YAP depletion and inhibition might be effective strategies to retard atherogenesis. Our results showed that MTX can suppress YAP activation and reduce the levels of inflammatory factors and adhesion molecules, (Figs. 2b, 3a, c), indicating that it might indeed be effective for the treatment of atherosclerosis. Both MTX and USS can cause an increase in p-YAP, but we did not observe a significant difference in p-YAP between USS and MTF + USS (Fig. 2c); we speculate that this is because it had already reached its maximum. Statins, which are widely used drugs that lower cellular cholesterol levels, have the ability to repress YAP activity and prevent YAP-mediated transcription [29]. Indeed, we observed that HUVECs treated with atorvastatin or MTX showed similar p-YAP protein levels. However, the exact molecular mechanism underlying these results should be evaluated in further studies.

Anti-inflammatory therapeutic strategies hold great potential for halting the progression and inducing the regression of atherosclerosis. MTX-loaded hybrid nanoconstructs target vascular lesions and inhibit atherosclerosis progression in ApoE^−/−^ mice [30]. MTX has been successfully used for the treatment of many immune or inflammatory diseases [31]. These positive effects of MTX on cardiovascular disease have also caught the attention of cardiologists. We previously found that MTX can reduce in-stent neoatherosclerosis in a rabbit model and that neoatherosclerosis frequently occurs at both edges of a stent affected by unidirectional shear flow [32], suggesting that local fluid shear stress is involved in in-stent neoatherosclerosis. Therefore, a better understanding of the effects of MTX on atherosclerosis under shear stress has clinical significance, and our experiment provides preclinical evidence that YAP/TAZ are potential therapeutic targets for in-stent neoatherosclerosis. However, the mechanism underlying the atheroprotective effect of MTX has not been reported. With respect to metabolic characteristics, MTX first inhibits AICAR transformylase and then results in AICAR accumulation and AMPK phosphorylation [17]. AMPK activity is associated with anti-inflammatory effects and exerts multiple protective effects during atherosclerosis [33, 34]. Several lines of evidence suggest that AMPK can inhibit YAP directly by phosphorylation of YAP and activation of the Lats kinase indirectly, resulting in YAP inactivation and causing their cytoplasmic retention and degradation [35]. Our data show that MTX treatment increased p-AMPK and p-YAP (Ser127) expression. Consistent with these findings, it is plausible that the AMPK pathway is responsible for MTX-mediated YAP (Ser127) phosphorylation based on our results indicating that the beneficial effects of MTX on DF-induced HUVECs are abolished by the knockdown of endogenous AMPKα. The protective effects of MTX were mediated, at least in part, by the suppression of AMPK activation. These results support the use of MTX for the treatment of atherosclerosis.

## Conclusion

From a therapeutic perspective, these findings provide insight into the mechanism through which MTX confers atheroprotection and might facilitate the development of new therapeutic approaches to limit atherosclerosis.

## Data Availability

All relevant data and materials are included in this published article.

## Abbreviations

YAP/TAZ: yes-associated protein and transcriptional co-activator with PDZ-binding motif
MTX: methotrexate
AMPK: adenosine monophosphate activated protein kinase
DF: disturbed flow
